# Resolution of long standing hypergammaglobulinemic purpura of Waldenström after initiating treatment for cystic fibrosis with Elexacaftor–Tezacaftor–Ivacaftor

**DOI:** 10.1016/j.jdcr.2023.09.037

**Published:** 2023-10-15

**Authors:** Kimberly Tantuco, Jennifer Beecker, Melanie Chin

**Affiliations:** a Department of Medicine, University of Ottawa, Ottawa, Ontario, Canada; b Division of Dermatology, the Ottawa Hospital, Ottawa, Ontario, Canada; c The Ottawa Hospital Research Institute, University of Ottawa, Ottawa, Ontario, Canada

**Keywords:** cystic fibrosis, Elexacaftor–Tezacaftor–Ivacaftor, hypergammaglobulinemic purpura, hypergammaglobulinemic purpura of Waldenström

## Introduction

Hypergammaglobulinemic purpura of Waldenström (HGPW) is a rare condition characterized by chronic recurrent purpura typically affecting the lower extremities, polyclonal gammopathy, elevated erythrocyte sedimentation rate (ESR), and elevated rheumatoid factor (RF). Limited data have been reported on HGPW in patients with cystic fibrosis (CF).^1^, ^2^, ^3^ We report a case of complete resolution of HGPW in a patient with CF soon after initiation of Elexacaftor–Tezacaftor–Ivacaftor (ETI).

## Case report

A 27-year-old woman with CF, severe lung disease, with chronic pulmonary *Staphylococcus aureus and Achromobacter xylosoxidans* infection presented with a 6-year history of recurrent purpuric rash of the lower extremities accompanied by pain and burning sensation, which were exacerbated by prolonged standing, walking, exercise, alcohol use, and tight clothing. Flares occurred twice a month lasting for approximately 7 days.

**Fig 1:**
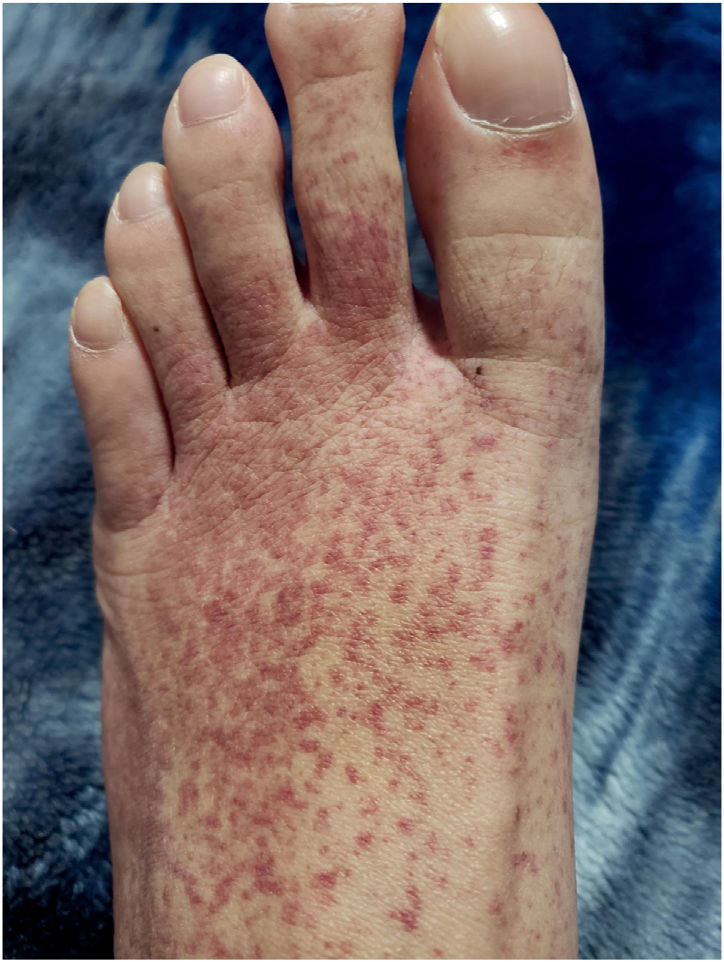
Purpuric macules dorsum aspect of the foot, clubbing.

**Fig 2:**
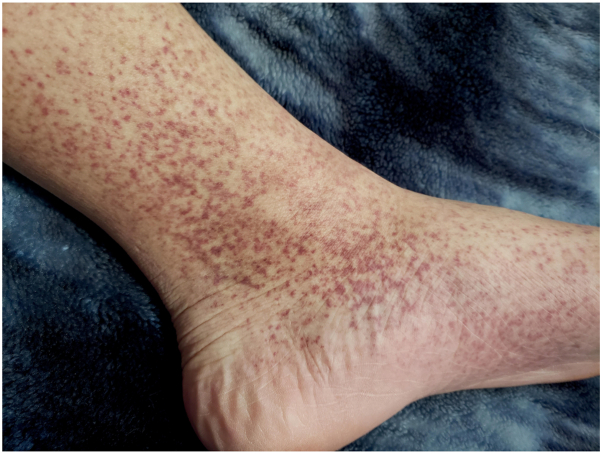
Hyperpigmented and purpuric macules on the instep and medial aspect of the lower leg.

On examination, hyperpigmented and small purpuric macules were noted over the lower legs and feet; clubbing with no obvious nailfold changes (Figs 1 and 2).

**Fig 3:**
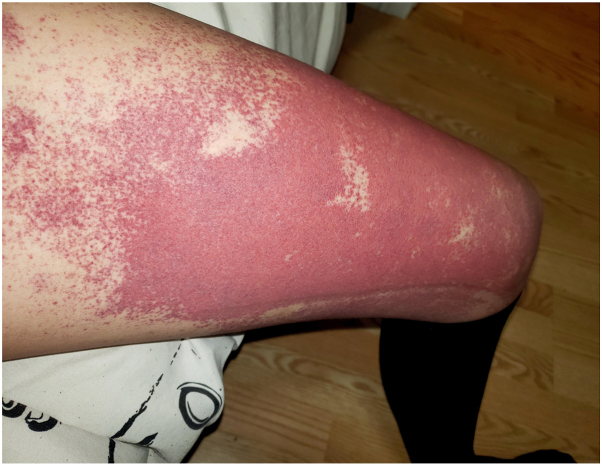
Flare of widespread purpuric macules and patches on the thigh.

Flares demonstrated widespread purpuric patches on the legs (Fig 3). Relevant investigations (Table I) revealed elevated levels of IgG, IgA, ESR, and RF. Skin punch biopsy was not done given this patient’s classic clinical presentation, and histopathologic examination is often nonspecific and time dependent.^1^

**Table I:** Laboratory data

White blood cells	9.2 x 10^9^/L (3.5-10.5)
Hemoglobin	110 g/L (115-155)
Platelets	420 x 10^9^/L (130-380)
ESR	**55 mm/h** (0-10)
Immunoglobulins	
IgG	**24.2 g/L** (7.0-16.0)
IgA	**5.7 g/L** (0.7-4.0)
IgM	0.9 g/L (0.4-2.3)
IgE	13.0 ug/L (≤240.0)
Serum protein electrophoresis	Diffuse polyclonal hypergammaglobulinemia
Gamma globulins	**22 g/L** (6-14)
Autoantibodies	
Antinuclear antibodies	**1:160 speckled**
Rheumatoid factor	**144 kIU/L** (≤14)

*ESR,* Erythrocyte sedimentation rate; *Ig,* immunoglobulin.

The patient was initially managed conservatively with compression socks and avoidance of precipitating factors. Hydroxychloroquine was added, which afforded minimal improvement over the next 2 years.

On December 3, 2021, ETI was started for her CF, which resulted in reduced daily pulmonary symptoms (cough, sputum production, and shortness of breath), reduced propensity for lung infections and increase in body mass index. Serendipitously, her skin symptoms cleared within approximately 30 days, and there has been no recurrence since.

## Discussion

HGPW is a rare condition first described by J. Waldenström in 1943.^2^ It is characterized by recurrent purpura affecting the lower extremities, polyclonal gammopathy, elevated ESR, and elevated RF.^1^^,^^2^ It can be either primary, or secondary and most commonly associated with Sjogren’s syndrome but has been reported secondary to autoimmune hepatitis, systemic lupus erythematosus, hepatitis C, CF, and multiple myeloma.^1^^,^ The link between these disease entities is polyclonal immunoglobin formation with immune complex deposits in blood vessels of the lower limbs.^1^

The purpura of HGPW is episodic and is frequently provoked by prolonged standing, exercise, tight clothing, and alcohol ingestion.^1^ It is more commonly seen over the lower extremities but can also involve the trunk and upper extremities.^1^ The purpura is commonly asymptomatic but can be itchy with burning sensation. In patients with CF, they may also develop edema of the feet, arthralgia, and arthritis.^3^ Purpura tends to fade within 2 weeks with residual hyperpigmentation, although recurrences are common.

Suggested laboratory work up of a patient with suspected HGPW includes a complete blood count, ESR, chemistry profile, total protein, urinalysis, quantitative serum immunoglobulins, serum protein electrophoresis, RF, antinuclear antibody, Sjogren's antibodies, cryoglobulins, rapid plasma reagin, tissue biopsy for light microscopy and immunofluorescence, and potentially other tests based on history and physical examination.^4^

HGPW has an indolent but recurrent course that can last for years.^5^ Treatment is not always necessary, and results may be disappointing. There is no current standard of treatment, options include avoidance of triggers, compression socks, corticosteroids, hydroxychloroquine, indomethacin, aspirin, colchicine, dapsone, mycophenolate mofetil, and rituximab.^1^^,^^3^

CF is a progressive multisystem disorder caused by the pathogenic mutations of the CF transmembrane conductance regulator (CFTR) gene.^6^ Although there is no cure for CF, major advances in understanding the CFTR gene have led to novel treatments with CFTR modulators.^7^ ETI is a combination drug that was approved by the US Food and Drug Administration in 2019 and targets the F508del mutation of the CFTR gene. Anticipated benefits from ETI includes improved lung function, decreased pulmonary exacerbations, increased body mass index, decreased sweat chloride, and a significant improvement in quality of life.^8^

CF-related HGPW is rare and was first described by Nielsen in 1978. More recently, Theisen et al^9^ reported on children ages 10 to 17 years old with concurrent CF and HGPW. The authors postulated that the development of HGPW in patients with CF is due to immune complex deposition in the setting of a large antigenic burden from recurrent infections. The combination of these disorders is associated with poor prognosis with a reported median survival of 16 months after the initial appearance of a rash.^3^ Although the purpura in CF-related HGPW tends to occur after the diagnosis of CF (and even late in the course of the disease), the purpura of non–CF-related HGPW can present several years prior to the diagnosis of the primary disease.^3^ Symptoms of CF-related HGPW tend to occur in the second or third decade of life, as was the case in our patient. Garty et al^10^ observed that in 15 cases of CF and purpura, the severity of the lung disease did not always correlate with the purpura. Although the authors noted that at least 50% of patients died within 2 years of the cutaneous purpura, there have been major advances in the treatment of CF since their study. Our patient remained clinically stable despite this disorder for approximately 6 years. Shortly after starting ETI, our patient experienced all the anticipated positive clinical effects in addition to complete resolution of her cutaneous symptoms. Emerging evidence has shown CFTR modulators to be effective beyond the pulmonary system.^7^ The mechanism to how ETI might be efficacious for HGPW in CF remains unknown. However, the authors postulate that the improvement of recurrent infections and decrease in inflammation, leading to less circulating immune complexes to deposit in the skin, may lead to improvement of concomitant purpura.

## Conflicts of interest

None disclosed.
